# Advances in the green synthesis and agrichemical applications of oxathiapiprolin derivatives

**DOI:** 10.3389/fchem.2022.987557

**Published:** 2022-08-29

**Authors:** Tingting Li, Jiamiao Jin, Jia Song, Jie Lv, Zhichao Jin

**Affiliations:** State Key Laboratory Breeding Base of Green Pesticide and Agricultural Bioengineering, Key Laboratory of Green Pesticide and Agricultural Bioengineering, Ministry of Education, Guizhou University, Guiyang, China

**Keywords:** oxathiapiprolin, oomycetes, structural modification, fungicidal activity, OSBPI

## Abstract

Oxathiapiprolin was developed with high antifungal activity and novel target protein and is used in the oomycetes control for crop protection. The structural modifications of oxathiapiprolin are summarized. The achievements and challenges in the structural modification of oxathiapiprolin are also discussed in this mini review. The outlook in this field is perspected according to our own opinion and understanding on the development of oxysterol binding protein inhibition fungicides.

## Introduction

**FIGURE 1:**
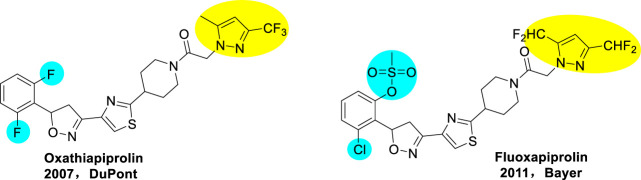
Commercial fungicides: oxathiapiprolin and fluoxapiprolin.

Oxathiapiprolin was developed as the first piperidinyl thiazole isoxazoline fungicide by Dupont in 2007 (Hanagan and Pasteris, 2009; Cristau et al., 2010; Pasteris, 2010), which was a new fungicide with novel chemical structure, containing pyrazole ring, thiazole ring, and isoxazoline ring (Figure 1). It is a class of oomycetes (Hardham, 2007; Kamoun et al., 2015) inhibitor, with the highest biological activity so far. It is also the most representative new pesticide in the 21st century and is expected to form a series of commercial products. Miao et al. (2016) demonstrated that the agent shows excellent biological activity against pathogens oomycetes. The EC_50_ and EC_90_ values of anti-oomycetes effect were 10^–4^ and 10^–3^ µg/ml, respectively, and there was no cross resistance with other oomycetes inhibitors. The agent was registered in China at the end of 2015 for the control of Phytophthora and downy mildew. In 2011, Bayer developed another piperidyl thiazole isoxazoline fungicide fluoxapiprolin (Tsuchiya et al., 2013) (Figure 1). These fungicides have great potential to control oomycetes for crop protection.

Due to the excellent biological activity and novel target proteins, many structural modifications of the oxathiapiprolin have been developed in recent years. Therefore, we consider that it is the right time to provide a systematic summary on the modification of this highly active fungicide. In this mini review, the structural modification and structure–activity relationship are discussed. A brief summary on the achievements and the challenges remained in the development of oomycetes fungicides is provided at the end of this review. An outlook into the future research direction within this field is also given based on our own opinion and knowledge on the trends of the development of oomycetes fungicides.

## Structural modification of commercial oxathiapiprolin

**FIGURE 2:**
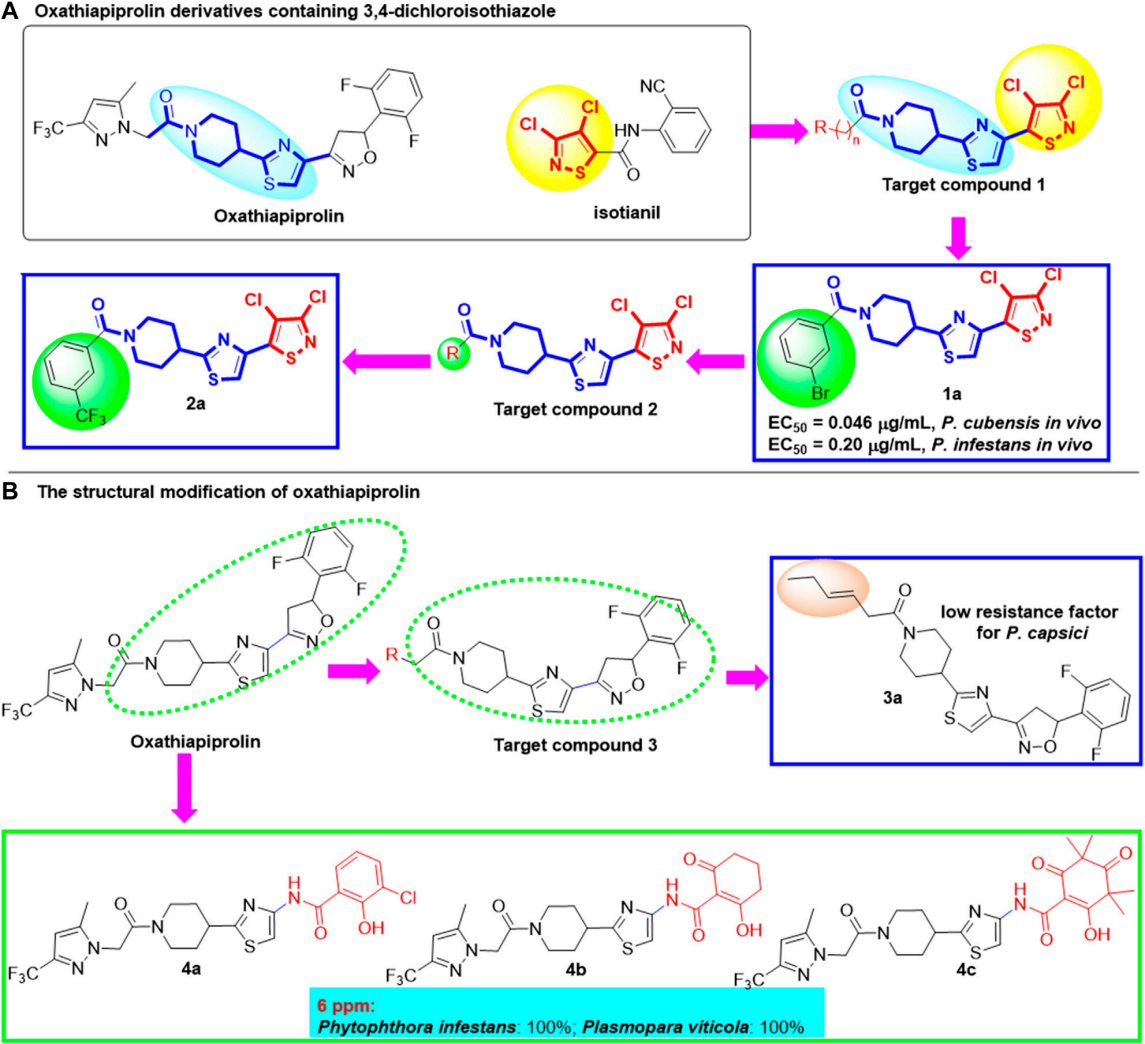
Terminal group replacement of oxathiapiprolin.

In 2018, in order to develop new fungicides with new mechanism of action and no resistance risk, Wu et al. (2018) designed and synthesized a series of novel target compounds based on commercial drugs oxathiapiprolin and isotianil and evaluated their antifungal and anti-oomycetes activities (Figure 2A). 3,4-dichloroisothiazole, which can induce the systemic acquired resistance (SAR), was introduced into the main scaffold of oxathiapiprolin. When *n* = 0 and R is *m*-Br phenyl group, compound **1a** shows excellent biological activity against *P. cubensis* and *P. infestans in vivo* with the EC_50_ values of 0.046 μg/ml and 0.2 μg/ml, respectively. At the same time, the expression of defense genes *npr1* and *pr1* was increased after treated with **1a** and infer that compound **1a** could induce SAR to improve the anti-oomycete activity *in vivo*. Subsequently, Wu et al. (2019) explored the substituents on the phenyl group of compound **2** in 2019 (Figure 2A). When the *m*-CF_3_ group was installed on the benzene ring, the corresponding compound **2a** exhibits better activity against *P. cubensis* and *P. infestans in vivo* at 1 μg/ml with the inhibitory rate of 100%, which is superior to that of oxathiapiprolin and isotianil. Although the field efficacy of compound **2a** against *P. cubensis* was 74.91% at 2 g ai/667 m^2^ in field efficacy trials, it can be used as a novel fungicide lead compound.

In order to find new compounds with low resistance factor for *P. capsici*, structural modification was carried out in 2020 by modifying the pyrazole ring (Wang et al., 2020; Wang Y. et al., 2022; Li et al., 2022) of oxathiapiprolin (Li et al., 2020), with most of the oxathiapiprolin skeleton remained unchanged. Compound **3a** possessed a low resistance factor for *P. capsici* and can be used as the lead compound for new fungicide development (Figure 2B). In 2015, Sulzer-Mosse et al. (2015) designed and synthesized a series of oxathiapiprolin derivatives and studied the structure–activity relationship. Compounds **4a**, **4b**, and **4c** exhibited excellent biological effects against phytopathogens *Phytophthora infestans* and *Plasmopara viticola*, with the inhibitory rate of 100% at 6 μg/ml. Meanwhile, structure–activity relationship studies revealed that a phenolic or enolic hydroxy function installed on the *β*-position of a carboxamide is significant for their bioactivities.

**FIGURE 3:**
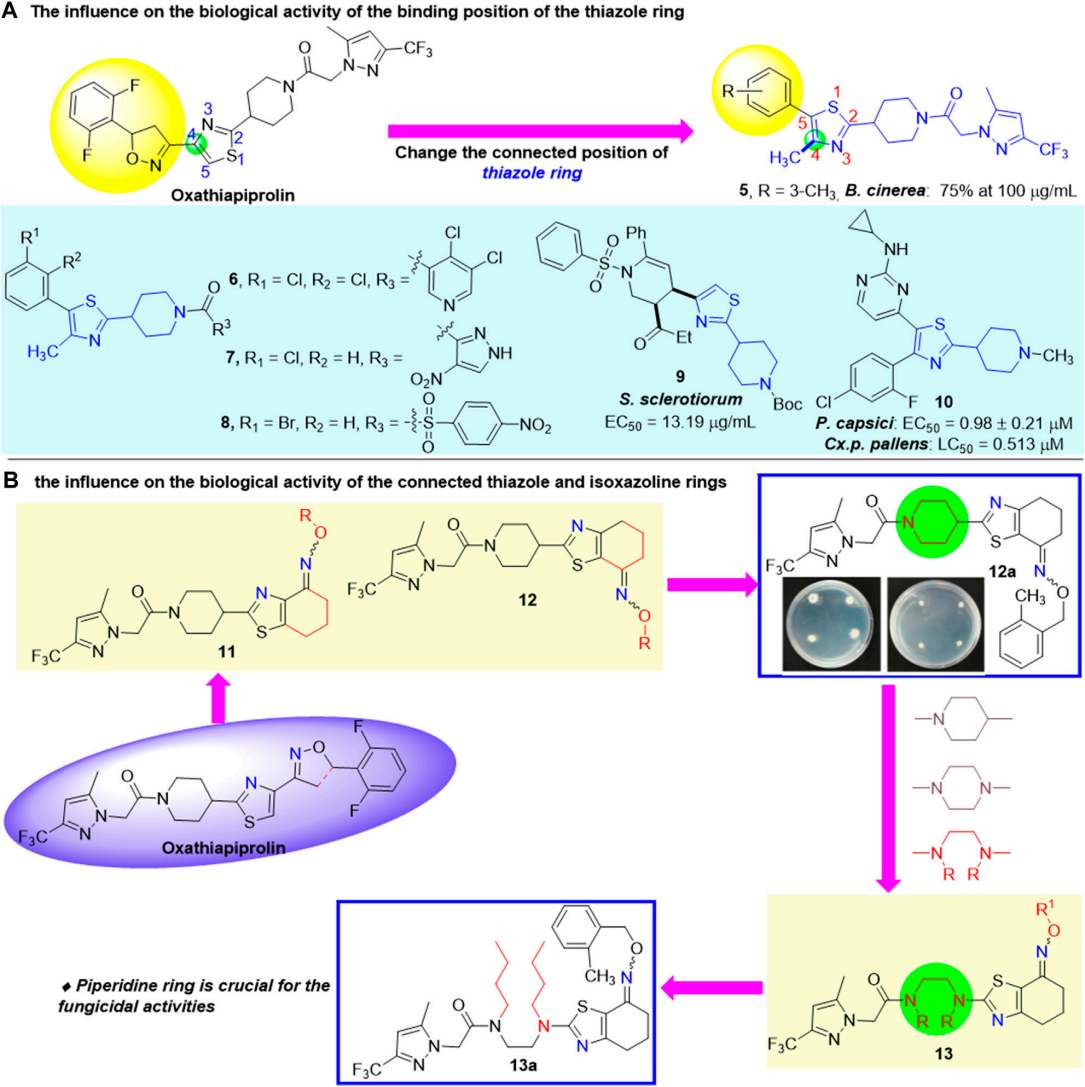
Modification of thiazole and piperidine rings in oxathiapiprolin.

The binding position of the thiazole ring (Wang M. et al., 2022) was also explored and proved to have significant influence on the biological activity. In 2019, Ding et al. (2019b)designed and synthesized a number of oxathiapiprolin derivatives (Figure 3A). The preliminary fungicidal activity showed that the inhibition rate of **5** against *B. cinerea* was 75% at 100 μg/ml. Other compounds exhibited poor fungicidal activities, which demonstrated that the thiazole ring is critical for the activity of oxathiapiprolin. Subsequently, when the terminal group was replaced and the remaining thiazole and piperidine rings in the oxathiapiprolin molecule unchanged (e.g., compounds **6**, **7**, **8**, and **9**), their fungicidal activities have not been significantly improved, instead of certain insecticidal activities (Zhu et al., 2016; Ding et al., 2019a; Ding et al., 2019c; Ding et al., 2020). However, Choi et al. (2015) reported a novel compound **10** with high fungicidal and insecticidal activities through the modification of the thiazole ring in 2015, with the EC_50_ value of 0.98 μM against *P. capsici* and the LC_50_ value of 0.513 μM against *Cx. p. pallens*.

The connected thiazole and isoxazoline rings existed in the oxathiapiprolin molecule are heteroatom-rich fragments bearing two nitrogen atoms positioned either at the same or opposite conformations. In 2021, Bian et al. (2021) revealed that the position of the two nitrogen atoms has significant influence on the bioactivity through the biological activity screening *in vitro* of novel piperidyl thiazole derivatives, containing oxime ether and oxime ester derivatives (Figure 3B). The bioassay results showed that the target compounds possessed moderate to good fungicidal activities against *P. capsici*. Compound **12a** exhibited the highest antifungal activity *in vitro* (EC_50_ = 0.0104 μg/ml), which was higher than dimethomorph (EC_50_ = 0.1148 μg/ml) and diacetylenyl amide (EC_50_ = 0.040 μg/ml). The activities of oxime ester compounds were lower than oxime ether compounds when the two nitrogen atoms are positioned on the opposite sides. Subsequently, based on the aforementioned results, a series of novel oxathiapiprolin derivatives containing oxime ether and oxime ester moieties were synthesized, in which the piperidine ring was opened (Tian et al., 2022) (Figure 3B). Their antifungicidal activities were evaluated and showed that the target compounds possessed moderate fungicidal activities against *Phytophthora capsici*, and the activities of these compounds were lower than that of oxathiapiprolin, suggesting that the piperidine ring was crucial for the fungicidal activities of these compounds.

## Conclusion and outlook

Oxathiapiprolin targets at the protein of oxysterol binding protein (OSBP) to give high fungicidal activity and have been used as the effective pesticide to control oomycetes for crop protection. It can be found that the piperidine and thiazole rings are crucial for the biological activity. Some heterocycles can be introduced into the structure to induce systemic acquired resistance and thus improve the *in vivo* control effects to various pathogens. Although a few achievements have been made, challenges still remain. The structural modification of oxathiapiprolin faces considerable limitations. All the derivatives disclosed to date showed lower activity than the commercial fungicide oxathiapiprolin. The introductions of additional bioactive functional groups and chiral fragments are promising strategies in the search for novel oxathiapiprolin-derived structures with greater fungicidal activities and lower risks.
